# The Honoured Dead

**Published:** 1887-08-27

**Authors:** O. W. Holmes


### THE HONOURED DEAD.

Take them, O Father, in immortal trust!
Ashes to ashes, dust to kindred dust:
Till the last angel rolls the stone away,
And a new morning brings eternal day.

O. W. Holmes.

